# Explanatory deep learning to predict elevated pulmonary artery pressure in children with ventricular septal defects using standard chest x-rays: a novel approach

**DOI:** 10.3389/fcvm.2024.1330685

**Published:** 2024-01-12

**Authors:** Zhixin Li, Gang Luo, Zhixian Ji, Sibao Wang, Silin Pan

**Affiliations:** Heart Center, Women and Children’s Hospital, Qingdao University, Qingdao, China

**Keywords:** artificial intelligence, pulmonary arterial hypertension, chest x-ray, ventricular septal defect, deep learning—artificial intelligence

## Abstract

**Objective:**

Early risk assessment of pulmonary arterial hypertension (PAH) in patients with congenital heart disease (CHD) is crucial to ensure timely treatment. We hypothesize that applying artificial intelligence (AI) to chest x-rays (CXRs) could identify the future risk of PAH in patients with ventricular septal defect (VSD).

**Methods:**

A total of 831 VSD patients (161 PAH-VSD, 670 nonPAH-VSD) was retrospectively included. A residual neural networks (ResNet) was trained for classify VSD patients with different outcomes based on chest radiographs. The endpoint of this study was the occurrence of PAH in VSD children before or after surgery.

**Results:**

In the validation set, the AI algorithm achieved an area under the curve (AUC) of 0.82. In an independent test set, the AI algorithm significantly outperformed human observers in terms of AUC (0.81 vs. 0.65). Class Activation Mapping (CAM) images demonstrated the model's attention focused on the pulmonary artery segment.

**Conclusion:**

The preliminary findings of this study suggest that the application of artificial intelligence to chest x-rays in VSD patients can effectively identify the risk of PAH.

## Graphical Abstract

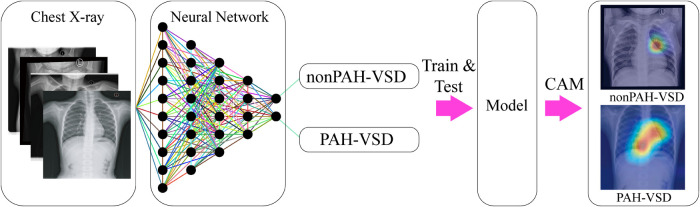

## Introduction

Among the growing population of adults with congenital heart disease, pulmonary arterial hypertension associated with congenital heart disease (PAH-CHD) is a major cause of increased mortality (1, 2). Ventricular septal defect (VSD) is the most common congenital heart anomaly (3). Complications such as PAH are common and require significant medical resources (4). The surgical outcomes of VSD repair are closely related to the age of the patient, with children under 2 years often experiencing a return to normal function post-surgery, while the long-term efficacy for those over 2 years with severe PAH remains uncertain (2, 5). It is widely accepted that repairing significant volume overload across the shunt between the systemic and the pulmonary circulations can lead to notable benefits and improve life expectancy in young patients (6). However, for VSD patients without early detection of PAH, even if the defect is successfully corrected, the mid to long-term outcomes after repair are unfavorable (7). Therefore, early detection of VSD patients with associated PAH (PAH-VSD) becomes a major clinical concern.

In pathophysiology, the development of PAH requires a genetic predisposition or other triggering factors that activate increased pulmonary blood flow, along with a series of mediators that cause vasoconstriction and vascular remodeling. Based on the pathological processes, scholars have identified various biomarkers that can indicate the risk of developing PAH-CHD such as Circulating endothelial cells (8), specific hyperoxia test (5), Acoustic (9), and surface electrocardiogram (10). However, current methods do not adequately utilize the information obtained from convenient standard chest radiographs to assist in diagnosis, such as increased lung markings and dilatation of pulmonary artery segments.

To fully explore the information derived from chest radiographs, machine learning and computer vision techniques offer methods to enhance insights, improve accuracy, and optimize the workload and time required for interpretation. The objective of this study is to develop and validate an AI-based interpretable prediction model that can predict the likelihood of PAH occurrence in children with VSD based on preoperative chest x-ray examinations. This research aims to leverage the power of artificial intelligence to identify high-risk children with PAH-VSD at an early stage, allowing for early prevention and better treatment, ultimately leading to improved patient outcomes.

## Materials and methods

### Data partition

This study is a retrospective analysis, and the chest x-ray images were collected from the Affiliated Hospital of Qingdao University for Women and Children. The inclusion criteria were as follows: (1) Children primarily diagnosed with ventricular septal defects upon admission; (2) Children without significant medical history; (3) Children who underwent both chest x-ray and echocardiography examinations after their initial hospitalization. The exclusion criteria were: (1) Low-quality data (lung lesions or air-trapping, and known history of lung disease or surgery.); (2) Outpatient cases with inadequate clinical information; (3) Patients with ventricular septal defects who only underwent either chest x-ray or echocardiography during their initial hospitalization; (4) Readmitted patients. To ensure the accuracy and reliability of the model, non-standard data were excluded. A total of 831 children who underwent their first diagnosis of ventricular septal defects and subsequent ventricular septal defect repair surgery from February 2015 to February 2023 were included for training and testing the predictive model using their chest x-ray images.

**Figure 1:**
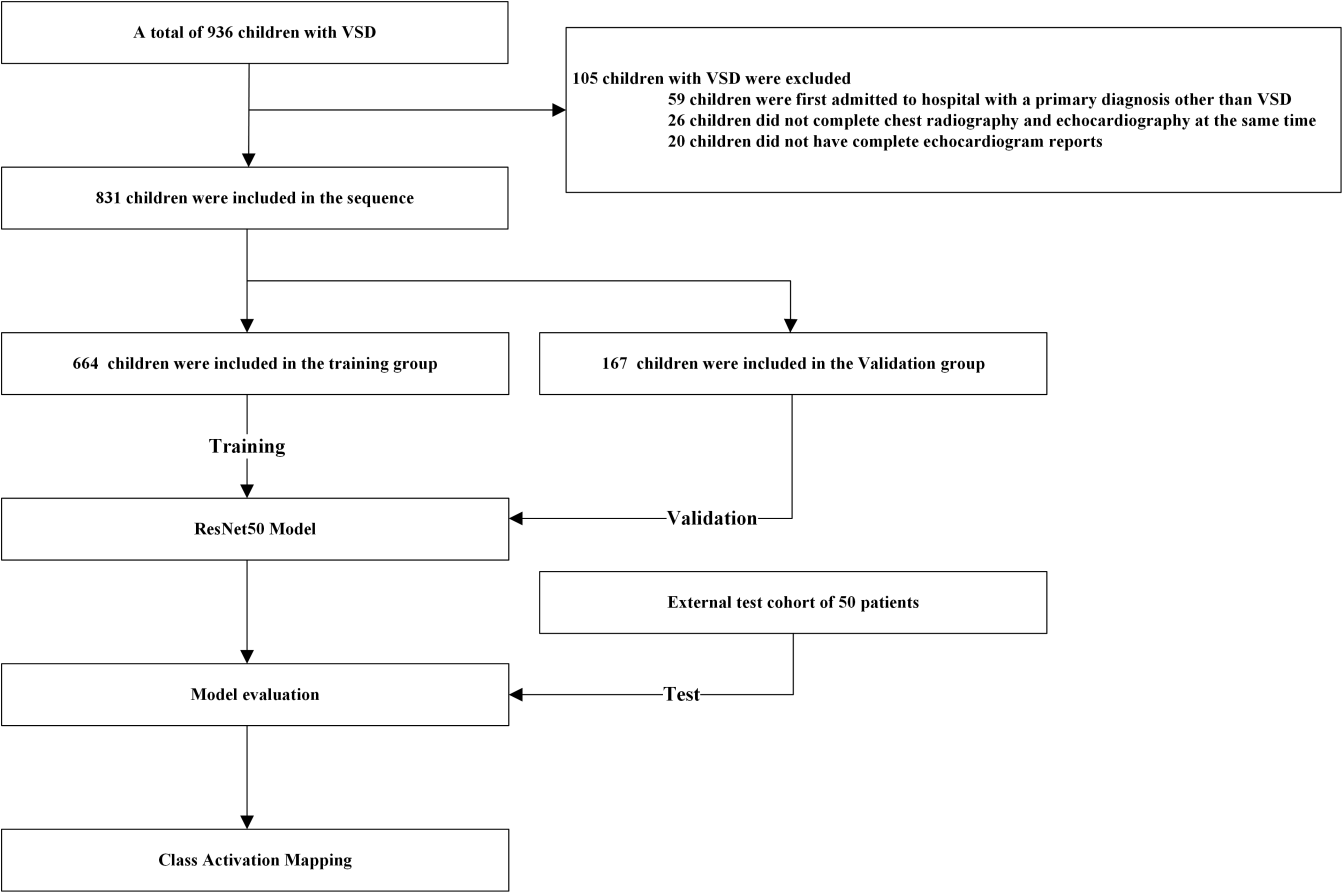
Flowchart of this study.

According to two pediatric cardiologists with over 10 years of experience, the children were divided into two groups based on their initial diagnosis confirmed by echocardiography reports (95%) or right heart catheterization (5%): the non-PAH group (670) and the PAH group (161). In this study, echocardiography was employed to explore pulmonary arterial hypertension, primarily utilizing pulmonary artery Doppler flow spectrum technology and the tricuspid regurgitation pressure gradient method, which has a high correlation with the clinically recognized “gold standard” right heart catheterization to measure pulmonary artery hypertension. The study protocol was reviewed and approved by our Institutional Review Board. As the data were obtained from patients who agreed to have their data comprehensively studied in routine clinical practice, informed consent was obtained from all patients. All methods adhered to the relevant guidelines and regulations (Figure 1).

### Image acquisition

In this study, conventional radiographs were acquired under standard conditions utilizing the DRX Evolution Plus (Carestream Health, USA). All DICOM images were converted to JPG images with a resolution of 256 × 256 through downsampling. The dataset was randomly divided into a training set (80%) and a test set (20%). Additionally, image augmentation techniques such as gamma correction, horizontal flipping, rotation, and pixel shifting were employed to enhance the images in the training dataset. Subsequently, resampling was performed to balance the data between the two groups, and hyper parameters were adjusted using grid search.

### Model development

We constructed a model based on ResNet50 to detect PAH in VSD patients. Residual neural networks help alleviate the vanishing gradient problem, allowing for the training of deeper networks (11). The network consists of 50 layers, divided into 5 blocks, each containing a set of residual blocks. Each residual block (RB) consists of two batch normalization layers, two rectified linear unit (ReLU) layers and two 3 × 3 convolutional layers. We then fine-tuned the pretrained model and performed nested ten-fold cross-validation. A batch size of 32 was set, and training was performed using the Adam optimizer. The network model was built using the PyTorch (version 1.6.0) deep learning framework on a computer equipped with a 16 vCPU AMD EPYC 9654 96-Core Processor and an RTX 4090 24GB GPU from NVIDIA Corp.

### Class activation mapping

We generated heat maps using class activation mapping (CAM) in order to identify regions with high activation levels (12–14). Global feature vector representation is obtained after the last convolutional layer. Convolutional Activation Mapping is utilized to generate high-resolution class-discriminative heat maps from the final convolutional layer. These heatmaps, generated by mapping the activations, are superimposed on the chest x-rays of patients diagnosed with VSD. This overlay provides an emphasis on the regions of utmost significance for predicting the PAH category. By utilizing CAM, decision makers can make the right decisions and gain a deeper understanding of the model (12).

### Role of the funding source

The study's funding source did not contribute to the study's design, data collection, analysis, interpretation, or manuscript preparation. The corresponding author had unrestricted access to all the data generated throughout the study and ultimately determined whether the manuscript would be published.

## Results

### Datasets

The training cohort consisted of 664 VSD patients. Participants were divided into the two groups: those with PAH (PAH group: 128 patients) and those without PAH (non-PAH group: 536 patients). There were no differences in the features between the training and validation cohorts. Clinical characteristics of the participants included in our study are presented in Table 1.

**Table 1:** Baseline characteristics of the study population.

	Training group	PAH-VSD group	Non-PAH VSD group	*P* value
Number	664	128	536	
Age, months	6 ± 1.3	6 ± 1.4	6 ± 1.2	>0.05
Male, *n* (%)	330 (49.5)	59 (46)	271 (50)	>0.05
Height, cm	65 ± 10	62 ± 12	66 ± 13	>0.05
Weight, kg	6.7 ± 2.5	6.6 ± 2.7	6.8 ± 2.3	>0.05
VSD diameter, mm	5.1 ± 2.7	5.5 ± 1.8	4.8 ± 3.5	>0.05
Number of TR (%)	176 (26.5)	45 (35%)	131 (24%)	>0.05

### Model evaluation

Trained artificial intelligence classification models distinguish unlabeled chest radiographic images into two groups: non-PAH and PAH. To evaluate the performance of our proposed model, we obtained different evaluation metrics such as accuracy, precision, sensitivity, and f1 score.

**Figure 2:**
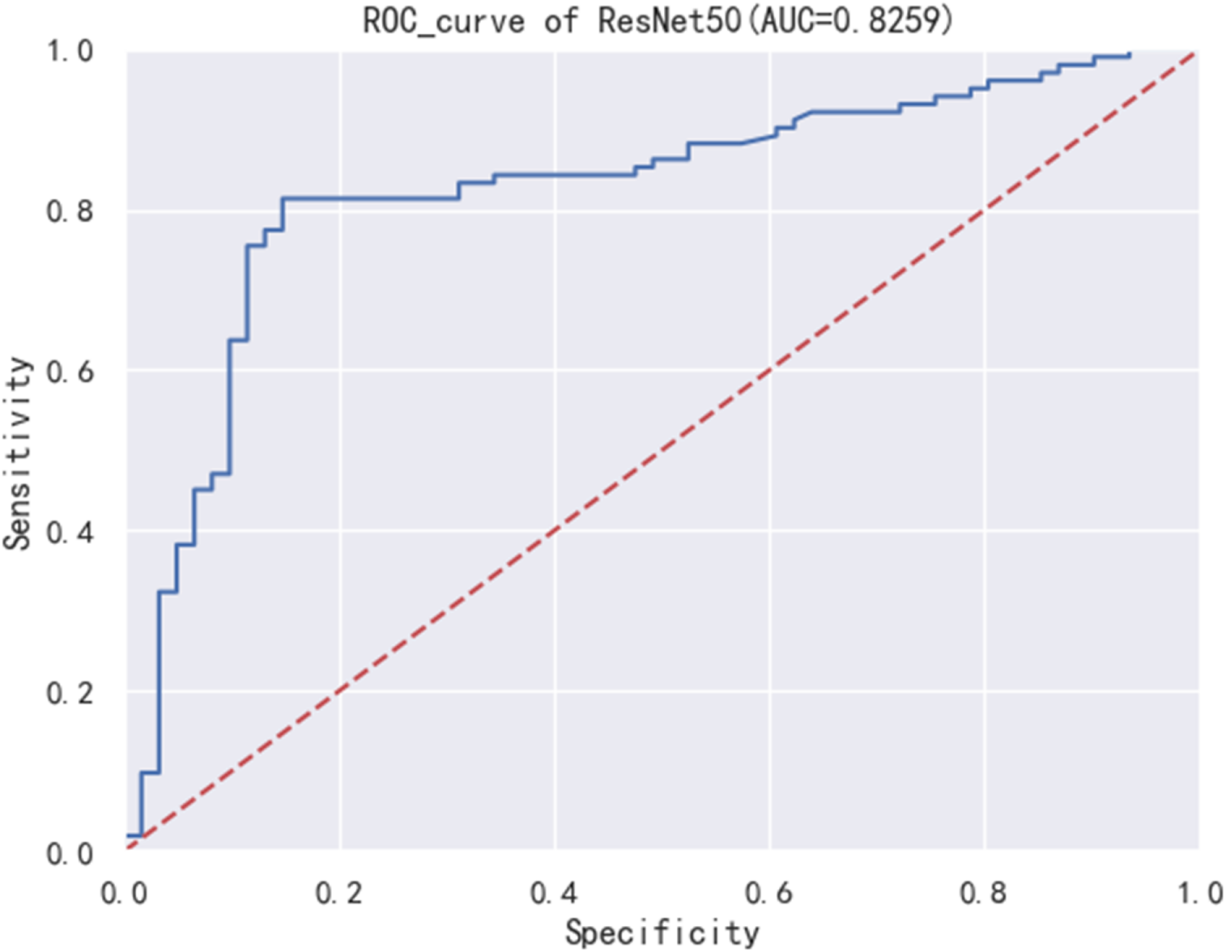
Validation group ROC curve of ResNet50 model.

**Figure 3:**
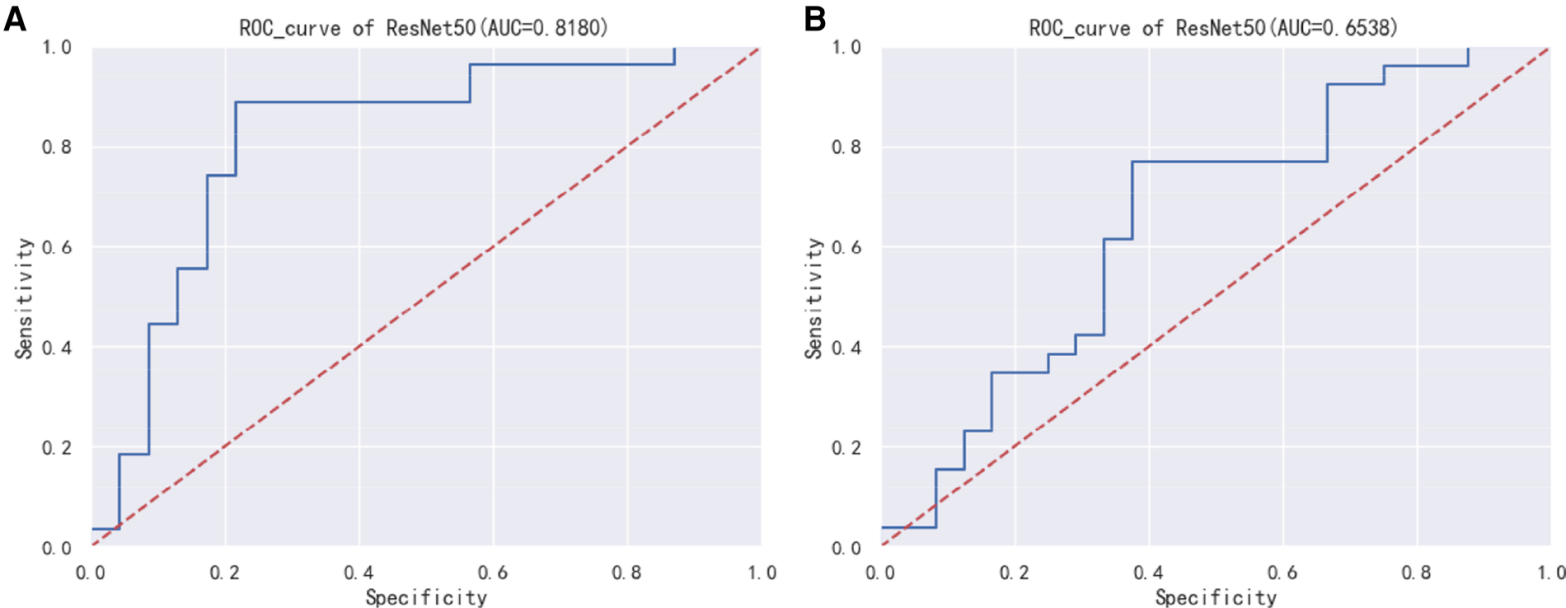
(**A**) ResNet50 model external test group ROC curve. (**B**) ResNet50 model human observers assess risk ROC curve.

**Figure 4:**
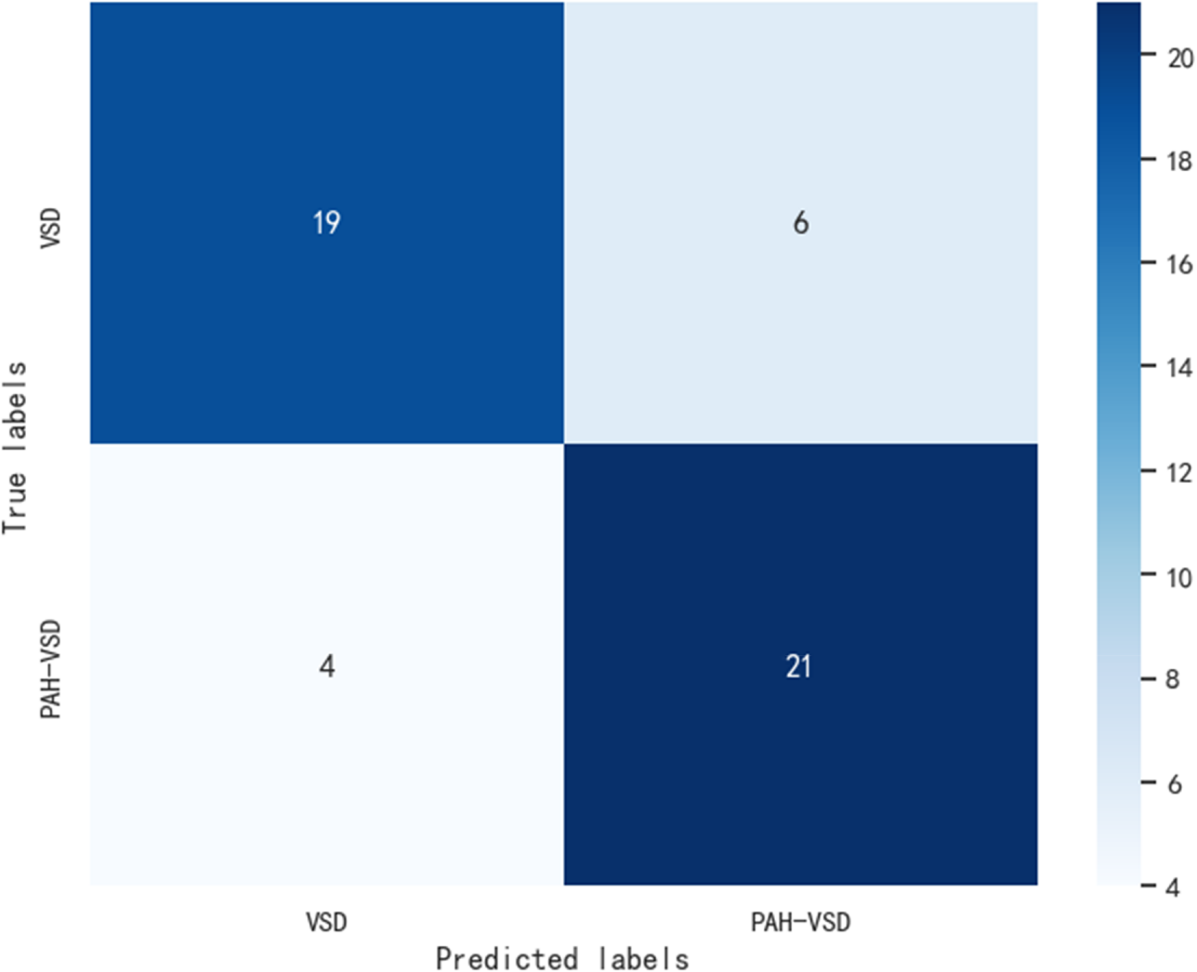
Resnet50 model external test group confusion matrix.

Figure 2 displays the Receiver Operating Characteristic (ROC) curve for the validation dataset, which serves as a graphical representation of the model's capacity to accurately discriminate between true positives and false positives. This curve provides an intuitive measure of the model's accuracy in differentiating between different classes. In order to evaluate the generalizability of our results, we collected an external test cohort consisting of 50 patients, for which the AUC of the DL algorithm was found to be 0.81. The AUC in this queue is similar to the original validation dataset queue, but significantly higher than the AUC of chest x-rays evaluated by human observers (0.65) (Figure 3). Figure 4 showcases the confusion matrix for the model's performance on the external test group. This matrix provides a comprehensive summary of the model's classification accuracy across all classes. The rows represent the predicted labels, while the columns represent the actual labels. To obtain a more comprehensive assessment of the model's accuracy, various performance metrics such as precision, accuracy, F1-score, etc., were calculated and presented in Table 2.

**Table 2:** The effect of the ResNet50 in external test group.

Model	Sensitivity	Specificity	Precision	Accuracy	F1 score	Matthews correlation coefficient
ResNet50	0.8261	0.7778	0.76	0.8	0.7917	0.6019

### Heatmap evaluation

**Figure 5:**
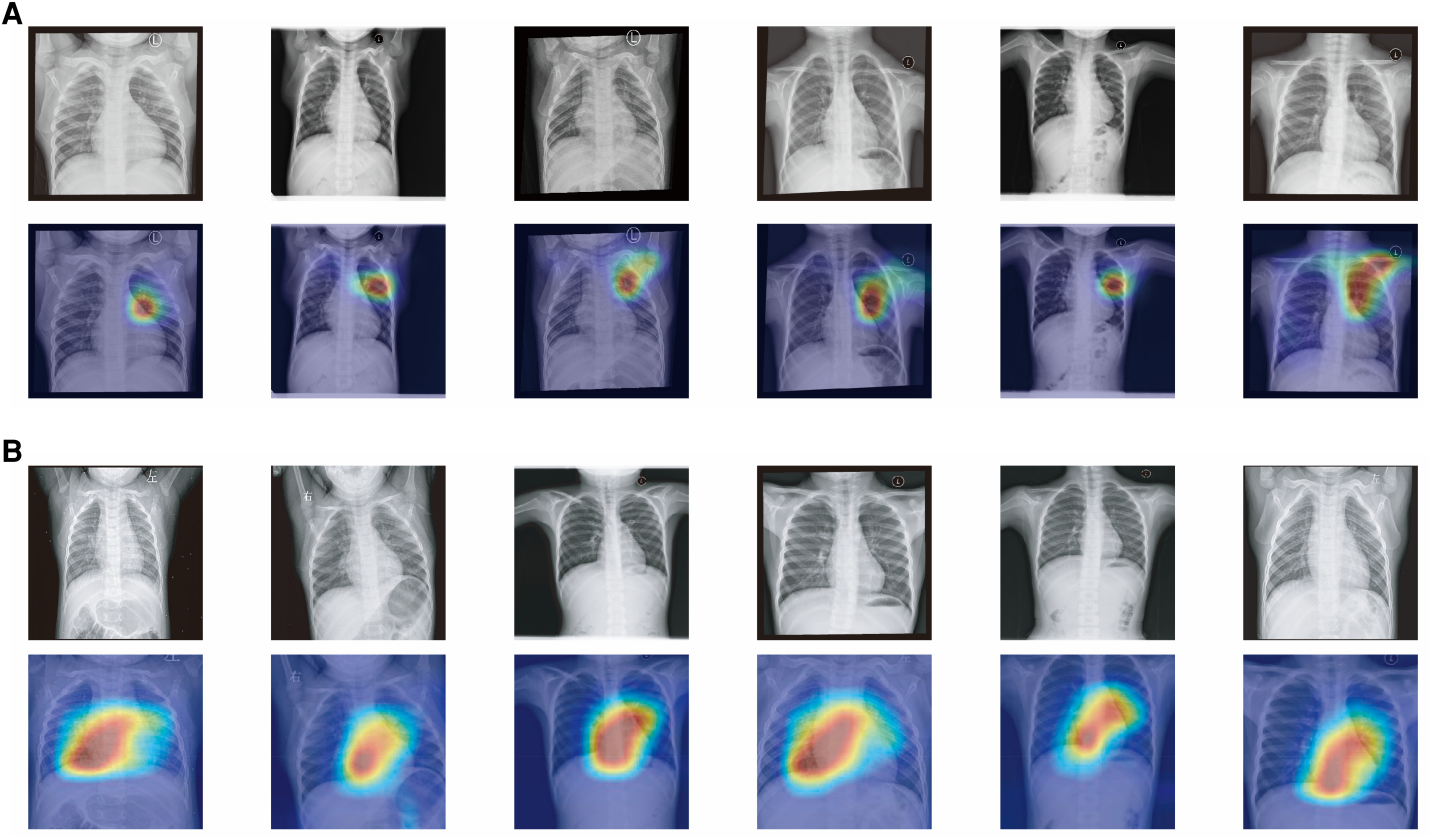
(**A**) Attention map of chest x-ray model for nonPAH-VSD patients. (**B**) Attention map of chest x-ray model for PAH-VSD patients.

In order to enhance the interpretability of clinical models and increase physicians' confidence in the models, we used Class Activation Mapping (CAM) to examine the attention focus areas in the images recognized by the artificial intelligence, which helped us determine the areas in CXRs that our model emphasized. Figure 5 exhibits heatmap overlays applied to 12 cases of true positive and true negative instances for the purpose of illustrating these regions of interest. Within the PAH group, the AI model demonstrates a tendency to concentrate on the pulmonary artery region as well as the right side of the heart. Meanwhile, in the nonPAH-VSD group, the AI model primarily focuses on the pulmonary artery. It is worth noting that characteristic features in chest radiographs of PAH-CHD patients include a prominent pulmonary artery segment and deepened pulmonary vascular shadow, indicating possible enlargement of the right atrium and right ventricle due to elevated pulmonary artery pressure. Hence, based on these observations, we are confident that our final AI model effectively discerns the variations in CXR images, which aligns well with our existing knowledge.

## Discussion

In this study, advanced deep learning techniques were used to develop a model for estimating the likelihood of PAH occurrence in patients with VSD through chest x-ray screenings. In the external test dataset, the most successful model exceeded an AUC threshold of 0.80, implying high sensitivity and specificity. In settings where alternative imaging modalities are not available, chest x-rays may be a crucial tool for detecting PAH patients, especially in settings where alternative imaging modalities are unavailable. Furthermore, these findings highlight the importance of machine learning-based approaches in healthcare, as they offer significant efficiency and accuracy improvements. Additionally, these results suggest that standardized and cost-effective examination methods can yield additional clinical information through AI algorithms. We believe this research represents the first attempt to predict PAH occurrence in VSD patients based on chest x-ray images using AI algorithms. As a result of CAM being incorporated into the model, clinicians will be able to recognize specific areas on chest x-ray images which indicate this disease's likelihood, which will provide further confidence to them.

### Clinical implications

Previously, CXR has been recognized as a useful tool in the examination of patients with elevated PAH, given its simplicity and cost-effectiveness, making it widely available worldwide. A recent study has shown that CXR measurements can identify a larger number of subjects with undiagnosed PAH. Furthermore, there are various methods for detecting PAH, including laboratory data, electrocardiography, and physical examinations, with AUC values reaching a maximum of 0.65. In previous studies, the limited availability of invasive data from right heart catheterization, which serves as the gold standard, has highlighted the potential superiority of our CXR-based model. From a reproducibility standpoint, automated evaluation for obtaining quantitative results without any user interaction, including measurements, is needed. Our results demonstrate that AI models can be trained to predict the occurrence of PAH in VSD patients based on CXR images. We believe that this study serves as a pilot investigation aimed at exploring the feasibility of applying deep learning algorithms to the clinical assessment of pulmonary arterial hypertension in VSD patients.

Our model can provide a highly explanatory and insightful tool due to the visual evidence supporting the classification results, which is an inevitable part of clinical diagnosis. By visually and accurately displaying significant regions of cardiac dysfunction, it can serve as an excellent artificial intelligence tool for radiologists in medical diagnosis, and can be extensively applied in clinical practices where comprehensive annotations are challenging to obtain.

### Limitations

Firstly, the sample size of patients in this study is limited. Deep learning algorithms require a large volume of data, typically thousands of patients, to achieve better generalization performance. Moreover, due to the small number of patients, we were unable to create models to predict specific types of pulmonary hypertension (such as preoperative PAH or postoperative PAH). Therefore, the development of viable AI models after classification was not feasible. Further evaluation through echocardiography and cardiac catheterization at referral centers is necessary. Additionally, as this study is retrospective in nature and the data collected were obtained from hospitals in the Eastern region of China, it is likely to have inherent biases. To address this issue, we plan to conduct a multicenter study involving multiple hospitals in the future. Given these limitations, this study is considered preliminary, and we believe that this report can serve as a motivating factor for future large-scale multicenter research.

## Conclusions

Applying artificial intelligence to CXR (a conventional, universal, and cost-effective test) is a potential tool for assessing the future risk of PAH in the VSD patient population. However, this preliminary study suggests that the use of artificial intelligence in CXR for predicting PAH risk in the VSD population is more effective than subjective judgments made by human observers. Nevertheless, the interpretability and stability of the results are still inadequate. It should be considered that it is premature to incorporate this technology into current guidelines.

## Data Availability

The original contributions presented in the study are included in the article/Supplementary Materials, further inquiries can be directed to the corresponding author.
